# Molecular Research in Multiple Sclerosis

**DOI:** 10.3390/ijms23052792

**Published:** 2022-03-03

**Authors:** Maurine Fucito, Damiana Pieragostino

**Affiliations:** 1 Center for Advanced Studies and Technology, University “G. d’Annunzio” of Chieti-Pescara, 66100 Chieti, Italy; 2 Department of Innovative Technologies in Medicine and Dentistry, University “G. d’Annunzio” of Chieti-Pescara, 66100 Chieti, Italy

The Special Issue, “Molecular Research in Multiple Sclerosis”, provides a better comprehension of the disease and establishes possible new biomarkers to ensure better care of MS patients in the future. It contains a review of the contribution of metabolomics in MS [1], the impact of SARS-CoV-2 infection [2], a review on common microRNAs (miRNAs) in MS and major depression (MD) [3], the first case of comorbid MS with Phelan McDermid Syndrome (PMcD) with regressive symptoms in a young woman who improved after treatment with methylprednisolone [4], and a review on intrathecal inflammation in progressive MS [5].

Molecular research includes the finding of biomarkers for diagnosis, prognosis, and therapy response monitoring of MS and its comorbidities. Such molecular changes are highlighted in the articles of this Special Issue as a means of providing better comprehension of the disease.

The first tool for better comprehension of MS is metabolomics [1]. The review on the contribution of metabolomics emphasizes that patients have different phenotypes at different time points. For diagnosis, higher scyllo-inositol and glutamine are reported in MS, while higher acetate, glutamate, lactate, and lysine are reported in neuromyelitis optica, once considered a form of MS. For prognosis, evidence of molecular mechanisms involved in MS is shown, such as oxidative/nitrosative stress, the kynurenine pathway from relapsing–remitting MS (RRMS) to chronic progressive MS, and eight biochemical pathways differences in CSF between RRSM and secondary progressive MS (SPMS). Additionally, in MS patients’ plasma, glycerophospholipids are found to be the most abundant, and a high body mass index is connected to an increase in ceramides. Regarding the treatment response, metabolomics are a useful tool for describing the response to different drugs such as interferon beta-1a and glatiramer acetate. Another interesting aspect of this review is the meta-analysis conducted to evaluate individual and group effects of metabolites upregulated in different fluids such as CSF, blood, and urine of MS patients and highlight pathways likely to play a role [1].

Data integration is a milestone in omics approaches, as demonstrated by Rispoli et al. [1] and Monaco et al. [5] in this Special Issue. By integrating results from metabolomics and neuroimaging data, Rispoli et al. showed a significant reduction in arginine plasma concentration in RRMS and a higher concentration of indolepyruvate in SPMS compared to RRMS. Also, a higher concentration of myelin basic protein, macrophage-derived chemokine, and 5.6-dihydroxyprostaglandin was highlighted in worse disease progression of SPMS [1]. In a similar approach, in Monaco et al.’s article, focusing on the intrathecal inflammation in PMS, neuropathology and molecular and MRI methodologies were integrated, showing molecules connected to a higher meningeal inflammation and grey matter demyelination, namely IFNγ, TNF, IL2, IL22, CXCL13, CXCL10, LTα, IL6, and IL10 [5].

Another important point in molecular research is the role of intrathecal inflammation compartmentalized to CNS and non-neural tissues in PMS [5]. Indeed, intrathecal inflammation is part of the clinical and pathological progression of MS, and there is a need to clarify how the compartmentalization of cellular inflammation occurs. In this review, the authors emphasize molecules connected to subpial lesions and CSF, meningeal, and choroid plexus inflammation. With these recent findings on the composition of CSF, cellular trafficking, and molecular exchange between compartments, the authors hypothesize that a proinflammatory milieu can damage the tissues being bathed. Further studies are needed to point out the mechanisms behind the promotion and maintenance of intrathecal inflammation [5]. In this context, we should mention a recent work showing that skull and vertebral bone marrow is a source of immune cells that accumulate in the dura without passing through the blood [6]. Also, intrathecal inflammation is thought to affect CSF flow rate and pulses. Interestingly, it has recently been shown that CSF flow is affected by the circadian cycle [7]. As the disruption of normal CSF circulation is thought to be connected to neurodegenerative diseases [8], there is an increased interest in understanding the mechanism of disrupted CSF flow rate and pulses by ongoing inflammation and whether it contributes to or clears brain inflammation.

Two papers highlight the fact that comorbidities must be considered for disease management [3,4]. Two comorbidities are discussed, namely MD [3] and PMcD [4]. First, MD is present in up to 50% of MS patients and shares abnormalities such as neuroinflammation, peripheral inflammation, gut dysbiosis, chronic oxidative and nitrosative stress, neuroendocrine abnormalities, and mitochondrial dysfunction. The analysis of miRNA regulation in those two diseases helps understand how the pathologies are connected and helps develop treatments that reduce depression in MS patients. Therefore, the author published a non-exhaustive table of 67 miRNAs and discussed their expression in MS and MD. As an example of the importance of considering MD morbidity in MS management, it was shown that IFN treatment has an incidence rate of depression greater than 0.1, probably due to the interaction between immune, endocrine, and neuronal pathways [3]. Second, the first case of comorbid MS in PMcD with regressive symptoms, in a young woman who improved after treatment with methylprednisolone, is discussed [4]. The goal of this paper is to evaluate if the incidence of PMcD and MS is coincidental or if there is a correlation in the pathophysiology, such as the involvement of SHANK3 and IGF1. More cases would be needed to confirm these hypotheses. However, this first case shows the importance of additional diagnostics in patients with PMcD with regressive symptoms [4].

Finally, another significant contribution to molecular research in MS is a review on the impact of SARS-CoV-2 infection to understand neurodegeneration processes and other common molecular pathways, such as vascular damage in MS [2]. This paper highlights how SARS-CoV-2 potentially induces demyelination in humans. In fact, the spike (S) glycoprotein from SARS-CoV-2 binds to receptor ACE2, which is highly expressed in respiratory epithelial cells and on neurons and glial cells. MS models induced by different viruses such as a murine coronavirus already exist, leading to demyelination in mice. In humans, the principal suspects are cytokine storms and the activation of immune cells, detailed by the authors. Also, the review emphasizes that, opposed to what was thought about a higher risk of infection in MS patients taking disease-modifying therapies (DMTs) with immunosuppressive effects, case reports did not describe an enhanced risk of hospitalization or fatal outcome. Surprisingly, some DMTs could even have a protective effect against the cytokine storm observed in COVID-19. Finally, the authors point out the enhancement of vascular damage in MS patients after SARS-CoV-2 infection. Three types of vascular dysfunctions are known in MS, namely cardiovascular incidents, chronic cerebral hypoperfusion, and chronic cerebrospinal venous insufficiency. Vascular damage in MS is connected to excessive blood platelets activity interacting with leukocytes and leading to an increase in autoreactive T cell infiltration in the CNS. Therefore, it increases the number of neuroinflammatory lesions. A strong expression of ACE2 in CNS is found in the perivascular astrocytes largely eliminated in MS, which should predict a low level of ACE2, therefore reducing the chance of the virus entering MS patients and decreasing the neurological complications. However, it is not possible to predict this using one factor (e.g., a decreased expression of ACE2) when several others have influence [2].

In conclusion, to date, few biomarkers are sensitive and specific enough to be used for population screening. Also, the sources of interindividual variability must be considered. MS is a complex and heterogeneous disease that integrates and crosses with other pathologies, generating unexpected effects, as in the case with MD and PMcD, and with infections such as COVID-19. Therefore, a single molecular marker is unlikely to exist, and efforts on *molecular research in multiple sclerosis* should be made towards integrating analytic approaches, including clinical characteristics, MRI variables and proteins, and metabolite concentrations to create signatures of MS disease diagnosis, prognosis, and treatment response.
